# The microbiome as a source of new enterprises and job creation: Considering clinical faecal and synthetic microbiome transplants and therapeutic regulation

**DOI:** 10.1111/1751-7915.12597

**Published:** 2017-01-04

**Authors:** Daniel van der Lelie, Safiyh Taghavi, Christopher Henry, Jack A. Gilbert

**Affiliations:** ^1^ Gusto Global LLC5960 Fairview RoadSuite 400CharlotteNC28210USA; ^2^ Argonne National Laboratory, Mathematical and Computer Sciences9700 S Cass AvenueLemontIL60439USA; ^3^ Computation InstituteUniversity of ChicagoChicagoIL60637USA; ^4^ The Microbiome CenterBioscience DivisionArgonne National Laboratory9700 S Cass Ave.LemontIL60439USA; ^5^ The Microbiome CenterDepartment of SurgeryUniversity of ChicagoChicagoIL60637USA

Modulation of the human immune system has become the focus for several novel approaches to treat conditions related to immune dysregulation, chronic infections and oncology. The human gut microbiome is being recognized as a key factor associated with the innate immune response, and exploring it has resulted in the identification of leads for therapeutics to treat conditions related to immune dysregulation and chronic infections, such as asthma, allergic rhinitis, eczema, IBD, IBS, Crohn's disease, chronic intestinal infections and various forms of food allergies like allergies to peanuts, shellfish and dairy products. A number of companies are active in this area and developing therapeutic faecal microbiome transplants [FMT (Van Nood *et al*., 2013)] and defined microbial consortia to treat infections with *Clostridium difficile*, of which the products of Rebiotix and Seres Therapeutics are among the most advanced. Overall, synthetic microbiomes (designer formulations) for transplants are preferred over FMT to attain desired/demanded standardization and safety standards, mode of action understanding and acceptability by regulatory agencies (Bojanova and Bordenstein, 2016). However, recent clinical trials to treat chronic infections with *C. difficile* using FMT were successful (Van Nood *et al*., 2013), while a defined microbial consortium performed below expectation. Therefore, additional research is required to better understand the critical roles and interdependencies of keystone strains in the human gut microbiome to design successful therapeutics.

The majority of designer formulations for modulating the immune response revolve around human‐derived butyrate‐producing bacterial species that belong to the *Clostridia* classes IV and XVIa to induce the accumulation of regulatory T cells that lead to the control of inflammation, a decrease in the secretion of a proinflammatory cytokine, or an enhanced secretion of an anti‐inflammatory cytokine by a population of human peripheral blood mononuclear cells. The best‐documented example of this approach is the work by Kenya Honda around a 17 species *Clostridium* strain consortium (Atarashi *et al*., 2011, 2013), VE202, which is currently being developed by companies like Vedanta Biosciences and Johnson & Johnson. Thus far, such probiotic formulations have proven useful in the treatment of immune disorders in only a subset of patients, further supporting the case for the need to better understand the interdependencies and interactions among keystone strains to improve engrafting and performance of probiotic formulations based on synthetic microbiomes.

The complexity of the human gut microbiome has limited the development of microbiome‐based therapeutics. This has prompted several efforts to develop predictive models to study the critical interdependencies of microbiome keystone species and the impact of host–microbiome interactions in specific diseases. Recent examples of such modelling systems include CASINO – Community and Systems‐level Interactive Optimization (Shoaie *et al*., 2015) – and AGORA – Assembly of Gut Organisms through Reconstruction and Analysis (Magnúsdóttir *et al*., 2016). It is expected that in the near future, predictive modelling will change the way microbiome research and development is being carried out, not just for microbial therapeutics, but also in adjacent areas, such as immunotherapy drugs for cancer treatment. Examples of processes, which are constrained by costs and time for experimental validation and will benefit from predictive modelling, include mode of action understanding, finding new indications, add‐on/adjunct therapies, root cause analysis, understanding of adverse events, optimized engraftment, effects of diet, secondary prevention (comorbidity), identification of predictive biomarkers, optimized trial design, detailed cohort studies and sample size extrapolation.

An example of a start‐up company that is at the forefront of using predictive modelling for every aspect of their R&D platform is Gusto Global: their modelling platform enables a significant (100‐fold or more) *in silico* reduction of experimental permutations for hypothesis‐driven experimental confirmation, mode of action understanding and product optimization. This provides a substantial opportunity for rapid optimization of existing microbial therapeutics through *in silico* modelling, as well novel therapeutic prediction. This is a rapidly developing area that requires a substantial focus on efficacy and reproducibility that will enable clinical application at scale.

## References

[mbt212597-bib-0001] Atarashi, K. , Tanoue, T. , Shima, T. , Imaoka, A. , Kuwahara, T. , Momose, Y. , *et al* (2011) Induction of colonic regulatory T cells by indigenous Clostridium species. Science 331: 337–341. 21205640 10.1126/science.1198469 PMC3969237

[mbt212597-bib-0002] Atarashi, K. , Tanoue, T. , Oshima, K. , Suda, W. , Nagano, Y. , Nishikawa, H. , *et al* (2013) Treg induction by a rationally selected mixture of Clostridia strains from the human microbiota. Nature 500: 232–236. 23842501 10.1038/nature12331

[mbt212597-bib-0003] Bojanova, D.P. , and Bordenstein, S.R. (2016) Fecal transplants: what is being transferred? PLoS Biol 14: e1002503. 27404502 10.1371/journal.pbio.1002503 PMC4942072

[mbt212597-bib-0004] Magnúsdóttir, S. , Heinken, A. , Kutt, L. , Ravcheev, D. A. , Bauer, E. , Noronha, A. , *et al* (2016) Generation of genome‐scale metabolic reconstructions for 773 members of the human gut microbiota. Nat Biotechnol. doi:10.1038/nbt.3703 [Epub ahead of print]. 10.1038/nbt.3703 27893703

[mbt212597-bib-0005] Shoaie, S. , Ghaffari, P. , Kovatcheva‐Datchary, P. , Mardinoglu, A. , Sen, P. , Pujos‐Guillot, E. , *et al* (2015) Quantifying diet‐induced metabolic changes of the human gut microbiome. Cell Metab 22: 320–331. 26244934 10.1016/j.cmet.2015.07.001

[mbt212597-bib-0006] van Nood, E. , Vrieze, A. , Nieuwdorp, M. , Fuentes, S. , Zoetendal, E. G. , de Vos, W. M. , *et al* (2013) Duodenal infusion of donor feces for recurrent *Clostridium difficile* . N Engl J Med 368: 407–415. 23323867 10.1056/NEJMoa1205037

